# Hidden features: exploring the non-canonical functions of metabolic enzymes

**DOI:** 10.1242/dmm.033365

**Published:** 2018-07-06

**Authors:** Peiwei Huangyang, M. Celeste Simon

**Affiliations:** 1 Abramson Family Cancer Research Institute, Perelman School of Medicine, University of Pennsylvania, Philadelphia, PA 19104, USA; 2 Departments of Cancer Biology, Perelman School of Medicine, University of Pennsylvania, Philadelphia, PA 19104, USA; 3 Cell and Developmental Biology, Perelman School of Medicine, University of Pennsylvania, Philadelphia, PA 19104, USA

**Keywords:** Cancer metabolism, Metabolic enzymes, Non-canonical functions

## Abstract

The study of cellular metabolism has been rigorously revisited over the past decade, especially in the field of cancer research, revealing new insights that expand our understanding of malignancy. Among these insights is the discovery that various metabolic enzymes have surprising activities outside of their established metabolic roles, including in the regulation of gene expression, DNA damage repair, cell cycle progression and apoptosis. Many of these newly identified functions are activated in response to growth factor signaling, nutrient and oxygen availability, and external stress. As such, multifaceted enzymes directly link metabolism to gene transcription and diverse physiological and pathological processes to maintain cell homeostasis. In this Review, we summarize the current understanding of non-canonical functions of multifaceted metabolic enzymes in disease settings, especially cancer, and discuss specific circumstances in which they are employed. We also highlight the important role of subcellular localization in activating these novel functions. Understanding their non-canonical properties should enhance the development of new therapeutic strategies for cancer treatment.

## Abstract (precis)

**Summary:** This Review summarizes recent findings about multifaceted metabolic enzymes with non-canonical activities outside their core biochemical functions, and how they may provide new therapeutic strategies for cancers.

## Introduction

Altered metabolism has long been observed in cancer cells (Warburg, 1956a,b). With the development of new experimental techniques, advances in cancer metabolism research have greatly enhanced our understanding of how cancer cells benefit from altered metabolism to support their growth. For example, subcellular fractionation revealed that the majority of key glycolytic enzymes are actually present in the nucleus (Kim and Dang, 2005). It is intriguing to speculate that these enzymes have unexpected nuclear functions, such as activating gene expression, which impacts specific cell decisions in response to fuel supply and demand. An emerging paradigm proposes that metabolic enzymes, rather than simply being components of biochemical pathways, are multi-functional proteins. They can act as mediators between growth stimuli, signaling pathways and downstream effectors, over and above the changes in metabolism, contributing to many other biological functions, such as gene transcription, apoptosis and cell cycle progression.

A key finding from studies of metabolic enzymes is the existence of mechanistic links between their nuclear localization and the regulation of transcription. By modulating gene expression, metabolic enzymes themselves facilitate adaptation to rapidly changing environments. Furthermore, they can directly shape a cell's epigenetic landscape (Kaelin and McKnight, 2013). Strikingly, several metabolic enzymes exert completely distinct functions in different cellular compartments. Nuclear fructose bisphosphate aldolase, for example, directly interacts with RNA polymerase III to control transcription (Cieśla et al., 2014), whereas, in the cytosol, it mediates signal transduction, vesicle trafficking and cell motility (Lincet and Icard, 2015). Indeed, a growing list of multifaceted enzymes supports the possibility that cells employ existing proteins in different and efficient ways, without the need to replicate or transcribe additional genes.

The aim of this Review is to provide an overview of metabolic enzymes for which non-canonical functions have been identified (Table 1) and to consider their implications in cancer. We begin by discussing how some metabolic enzymes translocate to the nucleus and regulate gene expression in different contexts, explaining how environmental cues are quickly relayed to regulate gene expression. We then describe the unexpected subcellular localization of several metabolic enzymes and their surprising roles in regulating major cell functions, such as cell cycle progression, DNA damage repair and apoptosis. We also review several unresolved questions about multifunctional enzymes and discuss their potential therapeutic implications in cancer.

**Table 1.**
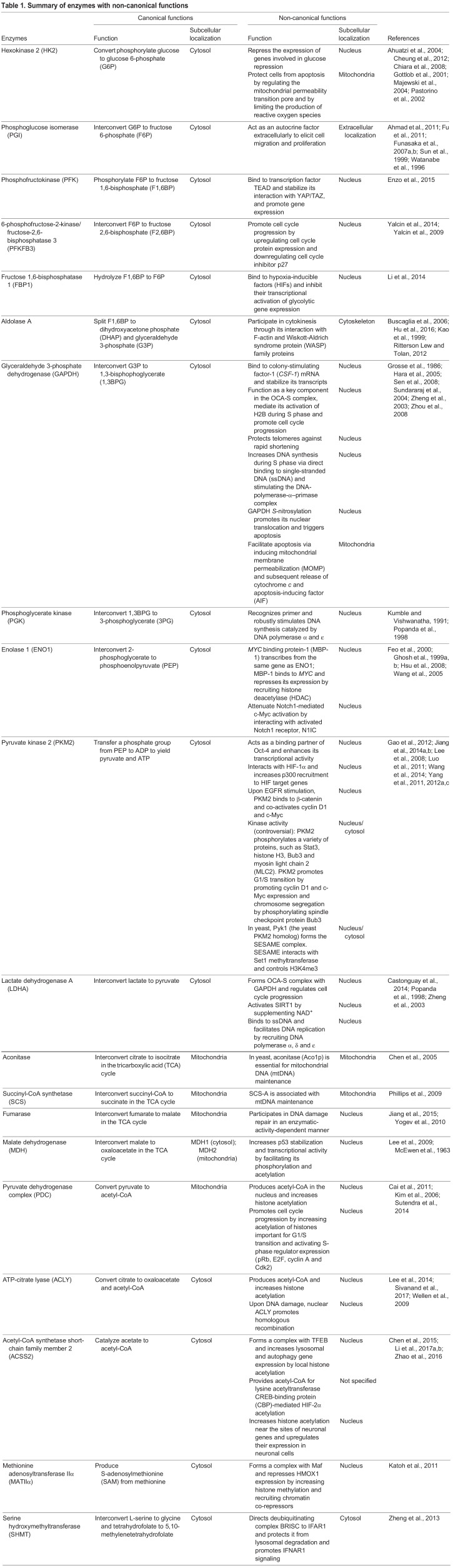
**Summary of enzymes with non-canonical functions**

## Regulation of gene transcription

A growing number of metabolic enzymes have been observed in the nucleus. It is reasonable to speculate that these enzymes are required for special nuclear functions, such as fundamental regulation of transcription and of the epigenome. Metabolic enzymes are highly sensitive to nutrient supply and demand, representing an efficient way to quickly establish adaptive responses by sensing metabolic stress and simultaneously modulating transcription. These enzymes participate in transcriptional control primarily by: (1) directly binding to target genes as transcription factors; (2) providing essential substrates for post-translational modifications; and (3) forming a transcription complex with other proteins. It is intriguing to propose that, in order to adjust to external cues, especially those driven by nutrient availability, cells coordinate their metabolic state and gene transcription through nuclear metabolic enzymes via complex mechanisms.

### Direct regulation

Many glycolytic (see Box 1 for a glossary of terms) enzymes are involved in transcriptional regulation by acting as transcription cofactors or by directly binding to DNA (reviewed in Boukouris et al., 2016; Yu and Li, 2017). The ability of metabolic enzymes to directly regulate gene transcription was first discovered in yeast, where glucose inhibits the expression of genes involved in its catabolism, a phenomenon called glucose repression. Glucose repression requires the presence of Hxk2 (HK2 in humans; see Box 2 for a list of abbreviations), a glycolytic enzyme that phosphorylates glucose to generate glucose-6-phosphate (Trumbly, 1992). Nuclear Hxk2 binds to the transcription factors Mig1 and Med8, and forms a complex with Tup1 and Cyc8 co-repressors, which binds to the promoters of Mig1 target genes, inhibiting their expression (Ahuatzi et al., 2004; de La Cera et al., 2002).

**Box 1. Glossary**

**Actin-myosin contractile ring:** a complex composed of filamentous actin (F-actin) and the motor protein myosin-2, along with additional structural and regulatory proteins. The contractile ring generates the constricting force for cytokinesis.

**Cell cycle checkpoint:** surveillance mechanisms in eukaryotic cells that monitor the order, integrity and fidelity of the major events of the cell cycle, therefore ensuring proper division of the cell.

**Cytokinesis:** the part of the cell division process during which the cytoplasm of a single eukaryotic cell divides into two daughter cells.

**Epigenetic modifications:** heritable chemical or physical changes in chromatin and DNA, such as DNA methylation, histone methylation and histone acetylation, which alter chromatin structures and consequently affect transcription and cellular functions.

**G1/S transition:** a stage in the cell cycle at the boundary between the G1 phase and the S phase, during which DNA is replicated.

**Gluconeogenesis:** a metabolic pathway that results in the generation of glucose from certain non-carbohydrate carbon substrates.

**Glycolysis:** a metabolic pathway that breaks down glucose into pyruvate, generating energy.

**The Warburg effect:** a shift from oxidative phosphorylation to glycolysis, even in the presence of oxygen, which has long been observed in cancer cells (Warburg, 1956a,b).

**Box 2. Abbreviations**

**53BP1:** p53-binding protein

**ACLY:** ATP citrate lyase

**ACS1:** acyl-CoA synthetase

**ACSS2:** acyl-CoA synthetase short chain family member 2

**AMPK:** AMP-activated protein kinase

**ATM:** ataxia telangiectasia mutated; a serine/threonine protein kinase

**BRCA1:** breast cancer type 1 susceptibility protein; a tumor suppressor

**BRISC:** BRCC36 isopeptidase complex

**Bub3:** mitotic checkpoint protein

**CBP:** CREB-binding protein

**Cdc25C:** M-phase inducer phosphatase 3

**Cdk1:** cyclin dependent kinase 1

**CSF**-1**:** colony stimulating factor 1

**DNA-PK:** DNA-dependent protein kinase

**EGFR:** epidermal growth factor receptor

**GAPDH:** glyceraldehyde-3-phosphate dehydrogenase

**HDAC:** histone deacetylases

**HK2:** hexokinase 2

**HMOX:** heme oxygenase

**IFNAR:** interferon-α/β receptor

**JMJD5:** Jumonji domain-containing protein 5

**LDHA:** lactate dehydrogenase A

**Mafk:** bZip Maf transcription factor protein

**MDH1:** malate dehydrogenase

**MLC2:** myosin light chain 2

**N1IC:** Notch1 receptor intracellular domain

**NADPH:** nicotinamide adenine dinucleotide phosphate

**NMDA:** N-methyl-D-aspartate

**NOS:** nitric oxide synthase

**NuRD:** nucleosome remodeling deacetylase

**OCA-S:** Oct-1 coactivator in S phase

**Oct:** octamer binding protein

**PcG:** polycomb-group protein

**PDK:** pyruvate dehydrogenase kinase

**PI3K:** phosphoinositide 3-kinase

**pRb:** retinoblastoma protein

**pVHL:** the von Hippel Lindau tumor suppressor protein

**SAICAR:** phosphoribosylaminoimidazolesuccinocarboxamide

**SAM:** S-adenosyl methionine

**SESAME:** serine-responsive SAM-containing metabolic enzyme complex

**Set1:** histone-lysine N-methyltransferase

**SNO-GAPDH:** *S*-nitrosylated GAPDH

**STAT3:** signal transducer and activator of transcription 3

**Swi/Snf:** SWItch/Sucrose non-fermentable; a nucleosome remodeling complex

**TAZ:** transcriptional coactivator with PDZ-binding motif (also known as WWTR1)

**TFEB:** transcription factor EB

**VDAC:** voltage-dependent anion channel

**WASP:** Wiskott-Aldrich syndrome protein

**YAP:** Yes-associated protein 1

**Fig. 1.**
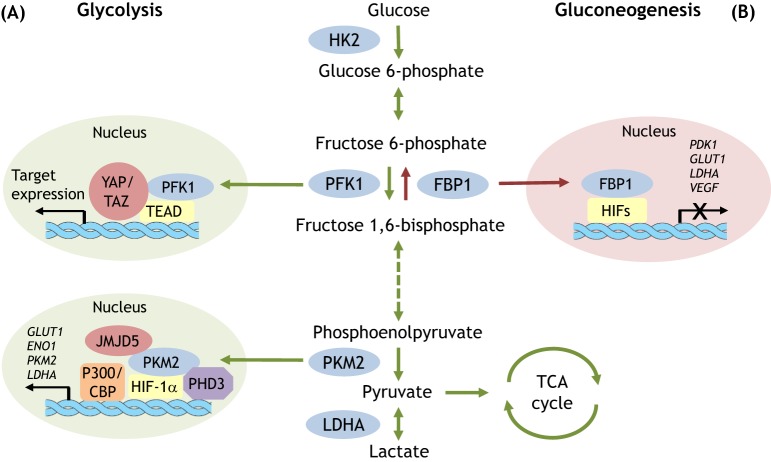
**Glycolytic and gluconeogenesis enzymes regulate opposing gene transcription.** Glycolysis and gluconeogenesis control glucose homeostasis. PFK1 and PKM2 are glycolytic enzymes that promote glucose catabolism, whereas FBP1 catalyzes glucose anabolism. PFK1, PKM2 and FBP1 can translocate into the nucleus and regulate the expression of target genes, including glycolytic genes, to support glucose metabolism accordingly. (A, upper) PFK1 binds to the transcription factor TEAD and potentiates its association with YAP/TAZ, leading to target gene expression. (A, bottom) JMJD5-PKM2 interactions facilitate the nuclear translocation of PKM2 to modulate the HIF-1α-mediated transcriptional reprogramming of metabolic genes. In addition, PHD3 enhances PKM2–HIF-1α binding and p300 recruitment to hypoxia response elements (HREs) in target genes. (B) By contrast, FBP1 colocalizes with HIF-1α and HIF-2α at target gene HREs and inhibits HIF transactivation. HK2, hexokinase 2; PFK1, phosphofructokinase 1; FBP1, fructose-1,6-bisphosphatase 1; PKM2, pyruvate kinase M2; LDHA, lactate dehydrogenase A; HIF-1α, hypoxia-inducible factor-1-alpha; JMJD5, Jumonji C domain-containing dioxygenase 5; CBP, CREB-binding protein; PHD3, prolyl-hydroxylase 3; TCA cycle, tricarboxylic acid cycle.

Subsequently, researchers identified additional examples of similar mechanisms of transcriptional regulation. Phosphofructokinase 1 (PFK1) irreversibly converts fructose 6-phosphate to fructose 1,6-bisphosphate, and its activity is subject to a variety of inputs, including the allosteric activator fructose 2,6-bisphosphate (F2,6BP) (reviewed in Mor et al., 2011). Besides its metabolic role, PFK1 appears to be involved in YAP/TAZ (Box 2) signaling. YAP and TAZ are key transcriptional coactivators that regulate organ size, cell proliferation and tumor metastasis (Harvey et al., 2013). The recruitment of YAP to its target genes is controlled by glucose metabolism; as such, enhanced glycolysis (Box 1) is associated with YAP activation. The connection between glucose metabolism and YAP/TAZ signaling is mediated by PFK1 (Enzo et al., 2015). In response to active glycolysis, PFK1 transits to the nucleus, binds to the transcription factor TEAD and stabilizes its interaction with YAP/TAZ in a catalytic-activity-dependent manner (Fig. 1). Through this novel nuclear role, PFK1 connects glucose availability and YAP/TAZ activity, fueling the proliferative functions of oncogenes in breast cancer cells. Glycolysis, in addition to its biochemical functions, also contributes to the regulation of YAP/TAZ activity and their ability to promote cell proliferation and tumorigenesis (Enzo et al., 2015). This finding highlights how cell metabolism influences signaling pathways.

Fructose 1,6-bisphosphatase 1 (FBP1) is a rate-limiting enzyme in gluconeogenesis (Box 1) and catalyzes the reverse reaction of PKF1. Mounting evidence suggests that FBP1 acts as a tumor suppressor in multiple cancer types (Chen et al., 2011; Dong et al., 2013; Hirata et al., 2016; Li et al., 2014). The transcriptional regulatory ability of FBP1 was first observed in clear cell renal cell carcinoma (ccRCC) tumors, in which FBP1 levels are uniformly decreased and FBP1 re-expression inhibits tumor progression by antagonizing glycolytic flux, thereby reducing the Warburg effect (Box 1) (Li et al., 2014). FBP1 also inhibits NADPH (Box 2) production from the pentose phosphate pathway, primarily by decreasing glucose uptake. Over 90% of ccRCC tumors harbor von Hippel-Lindau (*VHL*) mutations that stabilize hypoxia-inducible factors (HIFs) even under normoxia (Nickerson et al., 2008). However, in pVHL-expressing (Box 2) ccRCC cells, FBP1 no longer inhibits glycolysis and NADPH production, suggesting that HIFs are required for FBP1-mediated effects on glucose metabolism. Further investigation found that FBP1 physically interacts with both HIF-1α and HIF-2α, and that it suppresses HIF target gene expression by colocalizing with HIF-1α at hypoxia-responsive elements (HREs) within these loci (Fig. 1). Additionally, the suppression of HIF activity is abolished when a nucleus-excluded FBP1, but not the catalytically dead FBP1, is introduced into ccRCC cells (Li et al., 2014). These data suggest that FBP1's activity as a HIF transcriptional co-repressor is restricted to the nucleus in a catalytic-activity-independent manner.

Pyruvate kinase (PK) catalyzes the final step of glycolysis, converting phosphoenolpyruvate (PEP) to pyruvate and transferring a phosphate to ADP to produce ATP (Dayton et al., 2016b). Mammalian cells have four PK isoforms: two, PKM1 and PKM2, are splice variants transcribed from the same PKM locus (Noguchi et al., 1986). Unlike the constitutively active PKM1, the enzymatic activity of PKM2 is regulated by a variety of allosteric effectors, such as PEP, FBP, serine and SAICAR (Box 2), an intermediate in the biosynthesis of purines (Anastasiou et al., 2012; Chaneton et al., 2012; Christofk et al., 2008b; Keller et al., 2014). Recently, there has been a resurgent interest in PKM2 and its involvement in tumor progression (reviewed in Dayton et al., 2016b; Dong et al., 2016). However, its actual role in cancer progression remains controversial. Although some studies showed that PKM2 benefits tumor growth by increasing the flux through anabolic pathways (Christofk et al., 2008a; Lunt et al., 2015), others suggest that PKM2 is not an obligatory factor for tumor formation (Cortés-Cros et al., 2013; Dayton et al., 2016a; Israelsen et al., 2013). Strikingly, germline *Pkm2* deletion causes spontaneous formation of hepatocellular carcinoma (HCC), suggesting that a systemic disruption of metabolic homeostasis by PKM2 loss is sufficient for tumor initiation in a non-cell-autonomous manner (Dayton et al., 2016a).

In addition to its canonical role in glycolysis, PKM2 acts as a transcription coactivator in tumor cells (Lee et al., 2008; Luo et al., 2011; Wang et al., 2014). For example, it functions as an Oct-4 (Box 2) binding partner and enhances Oct-4-mediated transcriptional activity (Lee et al., 2008). Additionally, PKM2 antagonizes FBP1's inhibitory effects on HIF-1α by binding to HIF-1α and increasing p300 recruitment to the HREs of HIF-1α target genes, including *PKM2* itself, forming a positive feedback loop with HIF-1α (Luo et al., 2011). PKM2 also coactivates the expression of HIF-1α target genes that encode glycolytic enzymes, such as *GLUT1* and *LDHA* (Box 2), providing a mechanism by which PKM2 participates in a shift from oxidative phosphorylation to glycolysis under hypoxia. Interestingly, HIF-1α activation by PKM2 can be potentiated by JMJD5 (Box 2), a Jumonji C domain-containing dioxygenase. JMJD5 interacts with PKM2 and promotes its nuclear translocation, increasing the transcriptional activity of the PKM2–HIF-1α complex (Fig. 1) (Wang et al., 2014). As shown for human glioblastoma (GBM) cells, the transactivator property of PKM2 mediates crosstalk between EGFR (Box 2) and β-catenin signaling (Yang et al., 2011, 2012c). *EGFR* is frequently amplified in GBM, contributing to tumor development and progression (Verhaak et al., 2010). EGFR activation stimulates β-catenin signaling, another critical determinant of GBM progression. Upon EGFR activation, PKM2 translocates to the nucleus and binds to β-catenin, which then coactivates the transcription of cyclin D1 (*CCND1*) and c-Myc (*MYC*), resulting in cell cycle progression and tumorigenesis (Yang et al., 2011, 2012c).

Specific tricarboxylic acid (TCA) cycle enzymes are also involved in the transcriptional regulation of additional genes. The nuclear localization of MDH1 (Box 2) has been known for several decades (McEwen et al., 1963). Upon glucose deprivation, MDH1 translocates to the nucleus, increasing p53 stability and transcriptional activity by directly binding to p53 and facilitating its phosphorylation and acetylation (Lee et al., 2009). Thus, dysregulation of MDH1 in p53-mediated cell cycle arrest and apoptosis upon glucose depletion may increase cellular susceptibility to oncogenic transformation.

The notion that metabolic enzymes have DNA/RNA-binding properties suggests that they directly regulate gene expression (Grosse et al., 1986; Ronai et al., 1992). For example, due to GAPDH's (Box 2) predilection for AU-rich elements, it binds to the AU-rich terminal in the 3′ untranslated region of *CSF-1* (Box 2) mRNA (Zhou et al., 2008). GAPDH binding enhances *CSF-1* mRNA stability, increasing CSF-1 expression, which is an indicator of poor prognosis in patients with ovarian cancer. Although GAPDH has been considered as a housekeeping gene appropriate for use as mRNA and protein loading control in experimental settings, its expression in ovarian cancer is not consistent, with 50% of tumor specimens showing no GAPDH expression. More importantly, lower GAPDH expression correlates with reduced CSF-1 expression (Zhou et al., 2008), further supporting that GAPDH might regulate *CSF-1* gene expression.

Enolase 1 (ENO1) converts 2-phosphoglycerate to phosphoenolpyruvate and was also identified as a DNA-binding protein (Wang et al., 2005). Interestingly, the MYC promoter-binding protein-1 (MBP-1) is transcribed from the same *ENO1* gene, but in a shorter form (Feo et al., 2000). MBP-1 inhibits c-Myc expression via binding to the *MYC* promoter TATA-box sequence (Ghosh et al., 1999a; Ray et al., 1995). The recruitment of HDACs (Box 2) by MBP-1 might partially account for the transcriptional repression of c-Myc (Ghosh et al., 1999b). Furthermore, ENO1/MBP-1 interacts with the activated form of Notch1 receptor, N1IC (Box 2), and attenuates N1IC-mediated c-Myc activation (Hsu et al., 2008). Reciprocally, *ENO1* is a direct target of c-Myc (Kim et al., 2004); thus, it might function as a key player in the negative-feedback regulation of c-Myc-activated glycolysis. Importantly, reduced ENO1 expression is associated with poor prognosis of lung cancer patients (Chang et al., 2003). Moreover, forced MBP-1 expression results in impaired anchorage-independent growth *in vitro* and *in vivo* tumor formation in human breast carcinoma models, suggesting a tumor-suppressive role of MBP-1/ENO1 (Ray et al., 1995).

Taken together, the direct regulation of gene transcription by metabolic enzymes can be achieved through two mechanisms. Metabolic enzymes such as GAPDH and ENO1 function as transcription factors by directly binding to DNA. Conversely, enzymes without DNA-binding domains can interact with other transcription factors and alter their transcriptional regulatory ability, such as the interaction between HIFs and PFK1, FBP1 or PKM2. Notably, in the majority of cases, the enzyme's catalytic activity is not necessarily required for direct regulation of transcription. However, some metabolic enzymes indirectly regulate gene transcription through their reaction products in a catalytic-activity-dependent manner, which will be discussed later.

### Indirect regulation by protein modifications

Epigenetic modifications (Box 1) have important roles in genome-wide transcription. Growing evidence indicates that epigenetic modifications are sensitive to the metabolic status of cells (Cai et al., 2011; Shi and Tu, 2013) because they require substrates, such as acetyl and methyl groups, that are generated by metabolic reactions (reviewed in Kaelin and McKnight, 2013; Kinnaird et al., 2016; Lu and Thompson, 2012). Thus, metabolic enzyme ‘moonlighting’ in the nucleus provides a direct supply of otherwise unstable or impermeable metabolites for subsequent utilization by nuclear modifiers. In addition to providing essential substrates for epigenetic modification, metabolic enzymes can also modulate the activity of epigenetic enzymes. For instance, LDHA converts pyruvate to lactate and oxidizes NADH to NAD^+^, a key cofactor for the deacetylase SIRT1. LDHA physically interacts with and activates SIRT1 by supplementing NAD^+^, leading to enhanced histone deacetylation (Castonguay et al., 2014). This suggests that the indirect regulation of transcription by metabolic enzymes depends on their catalytic activity.

**Fig. 2.**
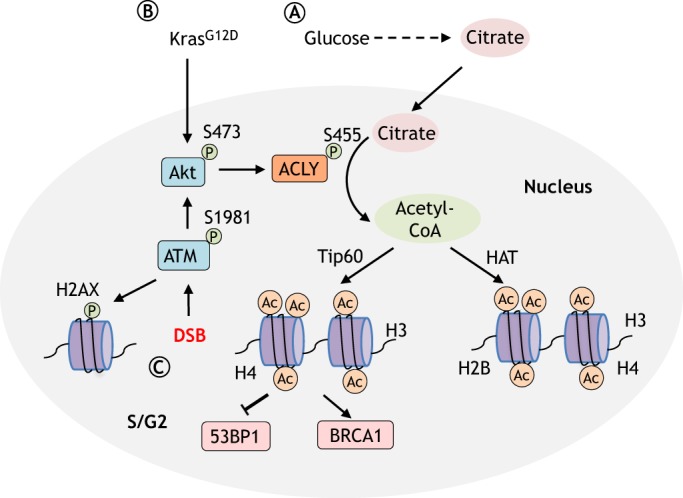
**Nuclear ACLY is involved in histone acetylation and homologous recombination.** Histone acetylation levels are affected by growth factor stimulation and nutrient availability. (A) ACLY converts glucose-derived citrate to acetyl-CoA, which is further used as a substrate for histone acetylation by HAT. (B) Oncogenic activation of Akt phosphorylates ACLY at site S455 and promotes ACLY-mediated acetyl-CoA production and histone acetylation. (C) ACLY also participates in homologous recombination (HR) after DNA damage. ATM-Akt-mediated ACLY activation is essential for histone acetylation at DSB sites mediated by Tip60 and for the subsequent recruitment of BRCA1 for DNA repair. Nuclear ACLY levels are highest during the S and G2 phases of the cell cycle, when repair by HR is preferred. Ac, acetyl group; ACLY, ATP-citrate lyase; HAT, histone acetyltransferase; DSB, double-strand break.

Histone acetylation is a dynamic process controlled by acetyltransferases and deacetylases, and involves the transfer of acetyl groups from acetyl-CoA. In mammalian cells, nuclear and cytoplasmic pools of acetyl-CoA can be produced by ACLY and ACS1 (Box 2). ACLY converts glucose-derived citrate to acetyl-CoA, whereas ACS1 uses acetate as its substrate. Both ACLY and ACS1 are found in the nucleus and cytoplasm, indicating that acetyl-CoA production occurs in both compartments (Wellen et al., 2009). Importantly, glucose addition, or the activation of oncogenic signaling, such as the Akt pathway, increases extramitochondrial acetyl-CoA pools in an ACLY-dependent manner (Fig. 2) (Lee et al., 2014; Wellen et al., 2009). As a consequence, ACLY is required for increases in histone acetylation in response to glucose availability and growth factor stimulation.

Although ACLY deletion impairs cell proliferation, cells remain viable by upregulating ACSS2 (Box 2) (Zhao et al., 2016). In contrast to ACLY-mediated global histone acetylation, ACSS2 tends to function locally (Li et al., 2017a,b). Recent studies have shown that, upon glucose deprivation, AMPK (Box 2) phosphorylates ACSS2 at the S659 site, tagging it for nuclear translocation. Nuclear ACSS2 forms a complex with TFEB (Box 2) (Li et al., 2017a,b). TFEB then guides this complex to the promoter regions of lysosomal and autophagy genes to promote their expression by local histone acetylation using acetyl groups produced by ACSS2. Notably, the acetate utilized by ACSS2 to generate acetyl-CoA is supplied by the deacetylation of histones and other nuclear proteins (Hallows et al., 2006; Li et al., 2017b). This suggests that, by recycling acetyl-CoA, nuclear ACSS2 is critical for cell survival and tumor growth under nutrient restriction. Similarly, in a neuronal cell culture model, ACSS2 levels increase in the nuclei of differentiating neurons, and functions as a chromatin-bound transcriptional coactivator that upregulates histone acetylation and expression of neuronal genes (Mews et al., 2017). Taken together, ACLY and ACSS2 are not just enzymes that provide acetyl-CoA for lipogenesis. Rather, they are sensors of nutrient supply and signaling pathways, and integrate nutrient metabolism to the regulation of histone acetylation and gene expression.

Similar to acetylation, histone methylation is closely connected with metabolic status, as histone methyltransferases use the one-carbon metabolism product, SAM (Box 2), as a methyl donor (Locasale, 2013). Methionine adenosyltransferase (MAT) converts methionine to SAM in an ATP-dependent manner (Sakata et al., 1993). MATIIα, one of three MAT isozymes, interacts with the transcription factor Mafk (Box 2), forming a complex (Katoh et al., 2011). Mafk contains a basic leucine zipper domain (bZip; DNA-binding domain) and activates or represses *HMOX1* (Box 2) expression, depending on its heterodimer partners (Hintze et al., 2007; Igarashi and Sun, 2006; Tahara et al., 2004). MATIIα also associates with the components of chromatin-related complexes, including Swi/Snf, NuRD and PcG (Box 2) complexes (Margueron and Reinberg, 2011; Wang et al., 2009; Wilson and Roberts, 2011), raising the possibility that MATIIα might locally provide SAM for histone methyltransferases. Indeed, MATIIα binds to the Maf recognition element in *HMOX1*, and its catalytic activity, as well as its interaction with chromatin co-repressors, is required for *HMOX1* repression. This study (Katoh et al., 2011) and the ACSS2 study mentioned above (Li et al., 2017b), indicate that localized increases in the metabolites used for histone acetylation and methylation, such as acetyl-CoA and SAM, might influence gene expression in response to the micro-environmental changes and the needs of the cell. As such, where and how the metabolic enzymes that generate these metabolites are recruited, and the epigenetic modifiers that they interact with, might be crucial for establishing the overall epigenetic status of a cell.

Metabolite availability also affects a range of protein modifications, including phosphorylation, acetylation and *S*-nitrosylation, which can be important for protein function. For example, nuclear ACSS2 is essential for the lysine acetyltransferase CBP (Box 2)-mediated HIF-2α acetylation in human Hep3B hepatoma cells and in the erythropoietin (EPO)-generating organs of hypoxic or acutely anemic mice (Xu et al., 2014). The hematopoietic growth factor EPO is encoded by a HIF-2α target gene (Gruber et al., 2007) and HIF-2α acetylation is essential for the efficient induction of *EPO* gene expression (Chen et al., 2012). In acutely anemic mice, acetate levels increase and serve as ACSS2 substrates, which are required for HIF-2α acetylation and subsequent *EPO* expression (Xu et al., 2014). This acetate–ACSS2–CBP–HIF-2α acetylation axis can also be found in cancer cells. In fibrosarcoma-derived HT1080 cells, ACSS2–HIF-2α signaling is activated in response to hypoxia and glucose deprivation. During such stresses, an increase in acetate correlates with HIF-2α acetylation and CBP–HIF-2α complex formation. Importantly, acetate supplementation promotes tumor growth and metastasis in an ACSS2- and HIF-2α-dependent manner (Chen et al., 2015). These data suggest that the acetate–ACSS2–CBP–HIF-2α acetylation axis links nutrient sensing and stress signaling in multiple disease settings.

In addition to its previously discussed transactivation roles, the protein kinase activity of PKM2 has also been proposed to regulate gene expression. PKM2 uses its substrate PEP as a phosphate donor for phosphorylation of a variety of factors, such as STAT3 (Box 2) (Gao et al., 2012), histone H3 (Yang et al., 2012b), Bub3 (Box 2) (Jiang et al., 2014a) and MLC2 (Box 2) (Jiang et al., 2014b). Its protein kinase activity may be required, as previously described, for the transcriptional coactivator function of β-catenin. PKM2 translocates to the nucleus with the help of importin α5 in response to EGFR signaling (Yang et al., 2012c), where it binds to and phosphorylates histone H3 at the T11 site. H3T11 phosphorylation displaces HDAC3 from the promoters of *CCND1* and *MYC*, promoting histone H3K9 acetylation and expression of c-Myc and cyclin D1, a regulator of the G1/S transition (Box 1) (Yang et al., 2012b). In addition to regulating cyclin D1 in this way, PKM2 directly controls cell cycle progression by binding to and phosphorylating the spindle checkpoint protein Bub3 (Jiang et al., 2014a). Bub3 phosphorylation is crucial for mitotic spindle assembly checkpoint, accurate chromosome segregation and cell proliferation (Sacristan and Kops, 2015). These results provide a mechanism where, in response to EGFR signaling, PKM2 promotes tumor cell proliferation by phosphorylating, and therefore activating, cell-cycle-related proteins. Strikingly, H3T11 phosphorylation by PKM2 seems to be conserved from yeast to humans (Li et al., 2015). Pyk1, the yeast homolog of PKM2, forms the SESAME (Box 2) complex with serine metabolic enzymes, SAM synthetases and an acetyl-CoA synthetase (Li et al., 2015). Glucose-derived serine promotes both H3K4 methylation and H3T11 phosphorylation catalyzed by SESAME complexes. SESAME interacts with the Set1 (Box 2) H3K4 methyltransferase complex at target gene promoters and provides SAM as the methyl donor for Set1. Then, Set1-mediated H3K4me3 facilitates PKM2-mediated phosphorylation of H3T11. Thus, by sensing glucose availability and glucose-derived serine, SESAME controls the crosstalk between histone methylation and phosphorylation, providing insights into the energy-sensing role of PKM2.

However, the proposed kinase function of PKM2 remains controversial because its kinase activity was not independently confirmed (Hosios et al., 2015). It was previously suggested that PKM2 phosphorylates proteins by using PEP as a phosphate donor. However, using [^32^P]-PEP as a tracer, labeling experiments failed to detect any proteins phosphorylated in a PKM2-dependent manner. Furthermore, direct transfer of phosphate from ATP to target protein by PKM2 was also not observed, arguing against a role for PKM2 as a protein kinase (Hosios et al., 2015). One possible explanation is that the observed phosphorylation of its protein targets, such as Bub3, is catalyzed by other protein kinases that use the ATP produced by PKM2 through its canonical pyruvate kinase activity. Future studies will delineate exactly how PKM2 regulates a long list of effector molecules.

Collectively, metabolic enzymes are able to regulate gene expression by altering histone and other protein modifications in response to fuel availability and signaling pathways.

## Components of protein complexes

A third way in which metabolic enzymes modulate gene transcription is by functioning as components of multi-protein complexes that recognize target gene sequences and anchor other regulatory factors. One such example is GAPDH, which is present in the Oct-1 coactivator complex, OCA-S (Box 2) (Zheng et al., 2003). OCA-S is a multicomponent transcriptional coactivator and a major determinant of *H2B* promoter activation during S phase (Luo and Roeder, 1995). GAPDH directly interacts with Oct-1 when it binds to the *H2B* promoter and anchors the OCA-S complex to Oct-1. GAPDH also has transactivation potential and may account partially for the transcriptional activity of OCA-S. Additionally, the NAD^+^/NADH ratio indicates the redox status of the cell, and an increased ratio enhances OCA-S transcriptional activity by increasing the interaction between GAPDH and Oct-1 at *H2B*. Together, these findings suggest a novel function of nuclear GAPDH and link cell metabolic stasis to *H2B* transcription and, consequently, the cell cycle.

Another example is serine hydroxymethyltransferase (SHMT), originally thought to act exclusively in one-carbon metabolism by catalyzing the conversion of L-serine to glycine and of tetrahydrofolate to 5,10-methylenetetraydrofolate. Surprisingly, SHMT has also been identified in a cytoplasmic deubiquitinating complex called BRISC (Box 2) (Zheng et al., 2013). BRCC36, a deubiquitinating enzyme that forms part of this complex, specifically targets lysine63-linked ubiquitin (K63-Ub) chains. SHMT directs BRISC to K63-Ub chains conjugated to IFNAR1 (Box 2), thereby protecting this interferon (IFN) receptor from K63-Ub-mediated internalization and lysosomal degradation. In turn, this enables IFNAR1-mediated signaling and maximal responses to IFN (Box 2) (Zheng et al., 2013). Unlike the other enzymes discussed above, SHMT regulates protein expression post-translationally in the cytoplasm. Notably, when associated with BRISC, the catalytic activity of SHMT is undetectable (Zheng et al., 2013), suggesting that SHMT plays a structural role rather than a catalytic role in the BRISC-SHMT complex.

In summary, an expanding list of metabolic enzymes present in the nucleus modulate gene expression, which suggests that metabolic adaptation to fuel availability may also be achieved by actively regulating gene transcription in addition to cytoplasmic metabolic reactions. In addition to controlling gene expression regulation, metabolic enzymes are also involved in multiple other biological processes.

## Other biological functions

The proper coordination of intracellular biological processes requires that the components and processes involved are separated into different subcellular compartments. For example, cytochrome *c* is an obligate mitochondrial protein involved in electron transport between complexes III and IV of the respiratory chain; once released into the cytosol, cytochrome *c* triggers apoptosis (Elmore, 2007). Some metabolic enzymes translocate between different cellular compartments to function in different contexts, often due to the membrane impermeability of their reaction products, such as nuclear ACLY and acetyl-CoA. In other cases, proteins might perform completely unrelated activities in different locations, increasing the functional options for the cell. GAPDH is one such multifaceted protein: it employs different mechanisms to regulate glycolysis, signal transduction in the cytosol, gene expression (Zhou et al., 2008), cell cycle progression (Zheng et al., 2003) and telomere maintenance (Sundararaj et al., 2004) in the nucleus, and apoptosis in both the nucleus and mitochondria (discussed later).

## Nuclear localization

Aside from interacting with nuclear proteins and DNA to affect gene transcription, several metabolic enzymes also participate in cell cycle regulation, DNA damage repair and apoptosis.

### Cell cycle regulation

Deregulation of the cell cycle underlies the aberrant proliferation characteristic of cancer cells, and loss of cell cycle checkpoint (Box 1) control promotes genetic instability. As discussed above, nuclear PKM2 promotes the G1/S transition and chromosome segregation (Jiang et al., 2014a; Yang et al., 2012b), whereas GAPDH contributes to cell cycle progression by acting as a cofactor of OCA-S during S phase (Zheng et al., 2003). Moreover, GAPDH increases DNA synthesis during S phase by directly binding to single-stranded DNA and stimulating the DNA-polymerase-α–primase complex (Grosse et al., 1986). Similarly, LDHA also binds single-stranded DNA (ssDNA) and facilitates DNA replication by interacting with DNA polymerases α, δ and ε (Popanda et al., 1998). Additionally, 3-phosphoglycerate kinase (PGK) acts as a primer recognition protein that robustly stimulates DNA synthesis catalyzed by DNA polymerases α and ε (Kumble and Vishwanatha, 1991; Popanda et al., 1998).

6-Phosphofructose-2-kinase/fructose-2,6-bisphosphatases (PFKFBs) are bifunctional enzymes that synthesize and degrade F2,6BP, a potent activator of PFK1. PFKFB isozymes are encoded by four genes, *PFKFB1-4* (reviewed in Ros and Schulze, 2013). PFKFB3 is distinguished by its presence in the nucleus and overexpression in human cancers, and is regulated by hypoxia and mitogens. The ectopic expression of PFKFB3 upregulates the expression of cell cycle proteins Cdk1 (Box 2), Cdc25C (Box 2) and cyclin D3, and downregulates the expression of the cell cycle inhibitor p27, leading to increased cell proliferation. The catalytic activity of PFKFB3 and its nuclear localization are both required for cell cycle regulation, indicating that the nuclear delivery of F2,6BP is essential for this function (Yalcin et al., 2009). Indeed, F2,6BP promotes Cdk1-dependent phosphorylation and the subsequent ubiquitination and proteasomal degradation of p27, which, in turn, de-represses the p27-mediated G1/S arrest (Yalcin et al., 2014).

The pyruvate dehydrogenase complex (PDC) was originally thought to convert glucose-derived pyruvate to acetyl-CoA solely in mitochondria. However, a recent study has shown that intact and functional PDC can translocate to the nucleus in a cell-cycle-dependent manner and produce a nuclear pool of acetyl-CoA (Sutendra et al., 2014). Nuclear PDC is implicated in cell cycle progression through two mechanisms. First, it regulates the acetylation of specific histone lysine residues that are important for G1/S phase progression, such as H3K9 and H3K18 (Cai et al., 2011). Second, the expression of S-phase regulators pRb (Box 2), E2F, cyclin A and Cdk2 is closely correlated with the level of nuclear PDC. Unlike mitochondrial PDC, which is inhibited by PDK (Box 2) (Kim et al., 2006), nuclear PDC is constitutively active due to the lack of nuclear PDK. Instead, nuclear PDC levels are controlled by growth factors or by the mitochondrial complex I inhibitor rotenone, suggesting that PDC might play a role in cancers that feature active proliferative signals and mitochondrial dysfunction.

Several studies have demonstrated that nuclear aldolase A is involved in cell division, in particular cytokinesis (Box 1) (Buscaglia et al., 2006; Kao et al., 1999). This function is probably achieved through its interaction with F-actin and WASP (Box 2) family proteins that regulate the polymerization of actin filaments (Buscaglia et al., 2006). Actin is a key component of the actin-myosin contractile ring (Box 1) (reviewed in Piekny et al., 2005). Aldolase A depletion increases the incidence of multinucleation, which is indicative of disrupted cytokinesis (Ritterson Lew and Tolan, 2012). Notably, in both mouse lung cancer cells and human squamous cell lung cancer cells, nuclear aldolase A is associated with increased proliferation, although its precise role remains elusive (Mamczur et al., 2013). Interestingly, the functional switch of aldolase A between cytoskeletal dynamics and glycolysis is regulated by PI3K (Box 2) signaling in epithelial cells. Upon stimulation by growth factors or insulin treatment, PI3K activates Rac, which in turn releases filamentous-actin-bound aldolase A by remodeling the actin cytoskeleton. Free aldolase A regains its catalytic activity and increases the flux through glycolysis (Hu et al., 2016). This study suggests a rapid and efficient mechanism for cells to increase the glycolytic flux by redistributing aldolase A. Moreover, it provides a mechanism whereby the master regulator, PI3K, coordinates cell metabolism, shape and function simultaneously.

### DNA damage repair

DNA damage repair is a genome-wide surveillance system that protects cells from potentially mutagenic DNA insults and maintains genomic integrity. When faced with double-strand breaks (DSBs), cells engage two main pathways for repair, non-homologous end joining (NHEJ), which is employed throughout the cell cycle, and homologous recombination (HR), which functions during the S and G2 phases of the cell cycle. 53BP1 and BRCA1 (Box 2) are important DNA damage repair factors that favor NHEJ and HR, respectively (reviewed in Panier and Boulton, 2014).

Accumulating evidence underscores the involvement of histone modifications in DNA repair (Yu et al., 2013). In particular, histone acetylation enables the repair machinery to access DSBs and recruit specific repair proteins (Gong and Miller, 2013). However, histone acetylation requires acetyl-CoA production in the nucleus, which mainly depends on the activity of nuclear ACLY (Wellen et al., 2009). Recently, Sivanand et al. reported that nuclear acetyl-CoA produced by ACLY participates in HR, adding further complexity to histone-acetylation-associated DNA repair (Fig. 2) (Sivanand et al., 2017). Upon exposure to ionizing radiation, ATM (Box 2) phosphorylates and activates Akt, which further promotes phosphorylation of nuclear ACLY at the S455 site. Of note, although cytosolic ACLY levels remain constant, nuclear ACLY levels increase in S/G2 phase and decrease in G1, suggesting that ACLY may be available to supply acetyl-CoA during S/G2 phase when HR is preferred. Phosphorylated ACLY increases acetyl-CoA production, which is essential for histone acetylation near DSBs, and the recruitment of BRCA1. Notably, although glucose availability affects acetyl-CoA production by ACLY, exposure to ionizing radiation does not alter glucose metabolism (Wellen et al., 2009), suggesting that a global reprogramming of glucose metabolism is unlikely to account for ACLY's role in DNA repair. This finding provides insights into how metabolic processes are actively integrated into cellular responses to DSBs, highlighting the importance of precise control of acetyl-CoA production in a spatial and temporal manner.

Histone methylation also plays an important role in regulating DNA repair. Fumarase (FH) catalyzes the reversible hydration and dehydration of fumarate to malate in the TCA cycle. FH is found in the cytosol and mitochondria of all eukaryotes (Kornberg and Krebs, 1957), but its nuclear localization was first discovered in yeast (Yogev et al., 2010). Upon DNA damage, yeast FH translocates to the nucleus and participates in repairing DSBs in an enzymatic-activity-dependent manner. The underlying mechanism involved has been revealed in human cells (Jiang et al., 2015). This study showed that DNA-PK (Box 2) phosphorylates FH at the T236 site, stimulating the local generation of fumarate near DSBs. Fumarate leads to increased levels of H3K36me2 through inhibition of the histone demethylase KDM2B, thereby recruiting DNA-PK at DSBs for NHEJ (Fnu et al., 2011). Notably, germline FH deficiency promotes susceptibility to hereditary leiomyomas and renal cell cancer (HLRCC) (Consortium, 2002; Launonen et al., 2001). One possible explanation is that a persistent lack of nuclear fumarate needed for DNA damage repair may render cells more sensitive to malignant transformation. A variety of studies propose other possible mechanisms for the increased susceptibility to oncogenic transformation in FH-deficient cells. In HLRCC, FH loss leads to an accumulation of fumarate, a competitive inhibitor of α-KG-dependent prolyl hydroxylase (PHD) that hydroxylates HIFs for degradation, thus activating oncogenic hypoxia pathways (Isaacs et al., 2005; Pollard et al., 2007). Other reports suggest that increased fumarate results in succination and inactivation of Keap1, abrogating its ability to repress the Nrf2-mediated antioxidant response pathway (Adam et al., 2011; Ooi et al., 2011; Sourbier et al., 2014). Together, nuclear ACLY and FH facilitate DNA damage repair by regulating histone modifications near the damage site, which recruit DNA damage repair proteins for HR or NHEJ.

### Apoptosis

The association between GAPDH and apoptosis was first established in neuronal cells, where the depletion of GAPDH completely blocks cytosine-arabinoside-induced apoptosis (Ishitani and Chuang, 1996). The nuclear translocation of GAPDH during apoptosis has also been demonstrated in various other cell systems (Dastoor and Dreyer, 2001; Sawa et al., 1997). The first question in uncovering the role of nuclear GAPDH in apoptosis is what mediates its nuclear translocation, since GAPDH lacks a nuclear-localization sequence (NLS). Hara et al. reported that cell stress activates NOS (Box 2), leading to the *S*-nitrosylation of GAPDH to generate SNO-GAPDH (Box 2), which interacts with the ubiquitin ligase Siah1. SNO-GAPDH and Siah1 then co-translocate into the nucleus. In turn, GAPDH stabilizes Siah1 and facilitates its E3 ubiquitin ligase activity, thereby promoting nuclear protein degradation (Hara et al., 2005). Nuclear GAPDH can be acetylated at the K160 site by p300/CBP via direct protein interaction, which in turn stimulates the acetylation and catalytic activity of p300/CBP. Consequently, p300/CBP activates its targets, such as p53, to trigger apoptosis (Sen et al., 2008). Interestingly, GOSPEL, a GAPDH-binding protein, is able to retain GAPDH in the cytosol by competing with Siah1 (Sen et al., 2009), therefore preventing GAPDH's pro-apoptotic activity. Additionally, GOSPEL overexpression protects neurons in culture and in mice from neurotoxicity elicited by excess activation of NMDA (Box 2), indicating a possible neuroprotective role of cytosolic GAPDH.

## Mitochondrial localization

### Apoptosis

The intrinsic pathways that initiate apoptosis are mitochondria-driven events (Elmore, 2007). Specific intracellular stimuli trigger the opening of the mitochondrial permeability transition pore (PTP), loss of mitochondrial membrane potential and release of two groups of pro-apoptotic proteins into the cytosol, including cytochrome *c* and apoptosis-inducing factor (AIF). Members of the Bcl-2 family of proteins, including the anti-apoptotic proteins Bcl-2 and Bcl-x, and the pro-apoptotic proteins Bax and Bak, regulate mitochondrial apoptotic events.

Interestingly, the glycolytic enzyme HK2 is a critical mediator of anti-apoptotic activity of Akt via its binding to VDAC (Box 2), a PTP component (Gottlob et al., 2001). The coupling of VDAC and HK2 was originally considered as a communication method between mitochondrial ATP synthesis and cytosolic glycolysis. In addition to its metabolic significance, this coupling serves as a downstream effector of Akt signaling and protects cells from apoptosis by inhibiting cytochrome *c* release in the presence or absence of Bax and Bak (Gottlob et al., 2001; Majewski et al., 2004; Pastorino et al., 2002). Moreover, mitochondrial HK2 inhibits apoptosis by regulating other PTP components, such as the adenine nucleotide translocator and cyclophilin D (Chiara et al., 2008), and by limiting the production of reactive oxygen species (ROS) (Cheung et al., 2012). Under hypoxia, the p53-inducible protein TIGAR binds to mitochondria and forms a complex with HK2, leading to a decrease of ROS levels and protection from cell death (Cheung et al., 2012).

In addition to its nuclear pro-apoptotic activity, mitochondrial GAPDH facilitates apoptosis by inducing mitochondrial membrane permeabilization (MOMP) and subsequent release of cytochrome *c* and AIF (Tarze et al., 2007). MOMP-induced caspase activation causes cell death; caspase-deficient cells cannot escape death but rather undergo a caspase-independent cell death (CICD). Paradoxically, GAPDH can protect cells from CICD and help them recover from MOMP (Colell et al., 2007). This is achieved either through the maintenance of mitochondrial potential via increased ATP production, or by a nuclear function associated with Atg12 that involves the autophagy-mediated clearance of defective mitochondria (Colell et al., 2007).

### Mitochondrial DNA maintenance

**Fig. 3.**
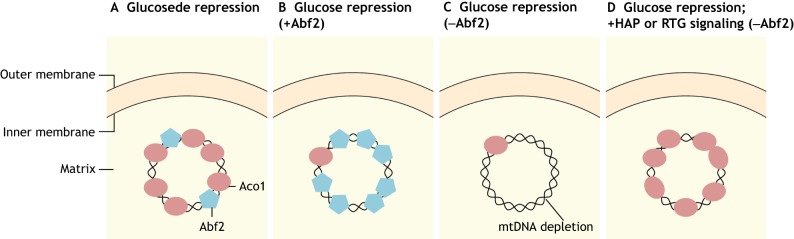
**Aconitase couples metabolic status to mitochondrial DNA maintenance.** Mitochondrial DNA (mtDNA) is packaged into protein-DNA complexes called nucleoids. The TCA cycle enzyme aconitase (Aco1) associates with nucleoids to stabilize mtDNA in response to changing cellular metabolism. In the presence of glucose, Aco1 expression is repressed, called ‘glucose repression’. (A) Under glucose de-repression, Aco1 is expressed and binds to mtDNA to form nucleoids. (B) Upon glucose repression, although Aco1 expression is inhibited, the mtDNA packaging protein Abf2 can replace Aco1 to bind and stabilize mtDNA. (C) Upon glucose repression, when Abf2 is deleted, mtDNA maintenance is reduced due to the lack of protecting proteins. (D) Even with glucose repression and Abf2 deletion, expression of the HAP2-5 transcription complex or Rtg1p and Rtg3p, which are components of mitochondria-to-nucleus retrograde signaling, restores Aco1 expression and the subsequent mtDNA maintenance. Aco1, yeast mitochondrial aconitase.

Mitochondria possess their own internal circular DNA genome (mtDNA), which encodes 13 essential subunits of the inner membrane respiratory apparatus (complex I, III-V) (reviewed in Scarpulla et al., 2012). Thus, mtDNA is essential for cells to maintain respiratory competency. Considering its significance, it is reasonable to speculate that certain mitochondrial proteins might be involved in protecting the integrity of mitochondria. Indeed, in yeast, the mitochondrial TCA cycle enzyme aconitase (Aco1p) can associate with protein-mtDNA complexes called nucleoids (Fig. 3) (Chen et al., 2005). Nucleoids incorporate proteins involved in mtDNA maintenance and transcription, and a range of signaling pathways controlling mitochondrial biogenesis, metabolism and retrograde regulation protein (RTG)-dependent retrograde mitochondria-to-nucleus signaling, whereby mitochondrial signals change nuclear gene expression (Gilkerson et al., 2013). Aco1p, an isomerase that converts citrate to isocitrate in the TCA cycle, is essential for mtDNA maintenance under different metabolic conditions, independently of its catalytic activity. The expression of Aco1p is controlled by many factors, including inhibitors such as glucose, and activators such as Huntingtin-associated protein (HAP2-5) and Rtg1p and Rtg3p. When glucose is available, Abf2p, an mtDNA-binding protein, can compensate for decreased Aco1p and maintain mtDNA stability due to its DNA-packaging function, whereas, in Abf2p-deficient cells, glucose repression of Aco1p can be rescued by HAP and RTG signaling (Chen et al., 2005). These observations suggest that nucleoid remodeling might be a strategy to maintain mtDNA integrity in response to cellular metabolism.

In parallel, a study of patients with encephalomyopathy identified a mutation in *SUCLA2*, the gene encoding the β-subunit of mitochondrial ADP-forming succinyl-CoA synthetase ligase (SCS-A) (Elpeleg et al., 2005). SCS-A deficiency is associated with mtDNA depletion; however, its subunits have not been identified in the mtDNA nucleoids required for mtDNA maintenance (Bogenhagen et al., 2003). Another plausible explanation is that insufficiency of mitochondrial deoxyribonucleoside triphosphate (dNTP) pools may cause defective mtDNA replication and, ultimately, mtDNA depletion. SCS-A is tightly associated with nucleoside diphosphate kinase (NDPK), which is crucial for maintaining the homeostasis of ribonucleotides and deoxyribonucleotides. Therefore, SCS-A deficiency may elicit mtDNA depletion syndrome due to a defect in the last step of mitochondrial dNTP salvage.

The discovery that multifaceted enzymes exert distinct functions in different cellular compartments suggests that metabolic enzyme translocation is spatially and temporally controlled to precisely accomplish specific cellular adaptations and fates. More work needs to be done to understand the complexity of the translocation mechanisms. Interestingly, phosphoglucose isomerase (PGI), in addition to performing intracellular metabolic functions, has an unexpected extracellular function, which will be discussed later.

## Extracellular localization

PGI catalyzes the conversion between glucose 6-phosphate and fructose 6-phosphate. Intriguingly, besides its role in glycolysis, PGI is also an autocrine motility factor (AMF) (Sun et al., 1999; Watanabe et al., 1996). Upon HIF-1α activation in tumor cells, PGI is secreted extracellularly and elicits cell migration and proliferation in an autocrine manner (Funasaka et al., 2005; Niinaka et al., 1998). PGI also promotes epithelial-mesenchymal transition (Ahmad et al., 2011; Funasaka et al., 2009, 2007b). Furthermore, the binding of PGI to the cell surface receptor AMFR results in increased migration, invasion and tumor angiogenesis (Lucarelli et al., 2015), and overexpression of PGI induces transformation and survival in NIH-3T3 cells by activating PI3K/Akt signaling (Tsutsumi et al., 2003). This oncogenic property of PGI may be achieved partly through its role in protecting cells from oxidative (Funasaka et al., 2007a) and ER (Fu et al., 2011) stress. Similar to enzymes that can translocate between different cellular compartments, we postulate that, to obtain extracellular functions, enzymes need to be secreted outside cells through either diffusion or via transporters located in the cell membrane.

## Therapeutic implications

The ‘hidden’ functions of metabolic enzymes are frequently exploited by cancer cells and thus present therapeutic opportunities. One strategy towards mitigating their cancer-promoting functions is to modulate their catalytic activity with small molecules. ACLY and ACSS2 inhibitors may suppress acetyl-CoA production in the nucleus, leading to decreased histone acetylation and gene transcription (Madeo et al., 2014). However, one issue with this strategy is that modulation of an enzyme’s catalytic activity may not fully block or activate its function, as some non-canonical functions are not dependent on enzymatic activity. For example, FBP1-mediated inhibition of glycolysis requires nuclear repression of HIFs independently of its catalytic activity (Li et al., 2014). Drugs that solely activate FBP1's enzymatic activity may not be sufficient to fully trigger its tumor suppressive function.

A second possible strategy is to target the subcellular trafficking of these enzymes, as a majority of their newly identified functions rely on specific subcellular localizations. For example, HK2 is anchored to mitochondria via VDAC and inhibits apoptosis by regulating the mitochondrial permeability pore and by limiting the production of ROS. Clotrimazole and bifonazole, which displace HK2 from mitochondria, can possibly restrain its protection of cells from apoptosis (Neary and Pastorino, 2013).

The third strategy is to block a catalytic enzyme's specific tumor-promoting features. One such example is PGI, the overexpression of which in various tumors negatively correlates with patients' clinical outcome (Dobashi et al., 2006). The autocrine capability of PGI underlines its potential as a circulating biomarker for cancer. Given that PGI increases cell migration and invasion via AMFR, the use of an anti-AMFR antibody has been shown to reduce the migratory and invasive capabilities in ccRCC (Lucarelli et al., 2015). Another example is PKM2, which has multiple, and not-yet clarified, roles in tumor progression. These are partly due to its pro-growth roles in the nucleus and its anti-growth activity in the cytosol. PKM2 forms dimers in the nucleus and tetramers in the cytosol. The small molecule TEPP-46 forces PKM2 to form tetramers, which decreases its recruitment to the nucleus and ultimately impairs H1299 human lung cancer cell xenograft growth (Anastasiou et al., 2012). In summary, the accumulating knowledge of non-canonical functions and regulatory mechanisms of metabolic enzymes may eventually implicate new molecular targets for cancer therapy.

## Concluding remarks

An increasing number of studies have discovered the unexpected features of metabolic enzymes that change classic concepts found in biochemistry textbooks. However, many questions remain unanswered. In this Review, we have discussed how metabolic enzymes translocate to different subcellular compartments and adopt non-canonical functions in response to different stimuli. Nevertheless, relatively little is known about the molecular mechanisms whereby these enzymes coordinate their canonical and non-canonical functions, as well as their subcellular distribution. Furthermore, nuclear translocation mechanisms are not fully understood, especially for those metabolic enzymes without NLSs. The nuclear import of those enzymes might depend on the chaperones of other nuclear shuttling proteins, such as in the co-translocation of Siah1 and GAPDH.

In conclusion, we are only beginning to elucidate the contributions that a broad spectrum of non-canonical functions of metabolic enzymes make to tumor progression. To obtain the full scope of non-canonical functions of metabolic enzymes, much more work remains to be done. Such advances in our knowledge will not only shed light on the comprehensive understanding of how metabolic enzymes orchestrate environmental cues and metabolic reprogramming, but also provide new avenues for therapeutic interventions in cancer and other diseases.
